# Antituberculosis activity, phytochemical identification of *Costus speciosus* (J. Koenig) Sm., *Cymbopogon citratus* (DC. Ex Nees) Stapf., and *Tabernaemontana coronaria* (L.) Willd. and their effects on the growth kinetics and cellular integrity of *Mycobacterium tuberculosis* H37Rv

**DOI:** 10.1186/s12906-017-2077-5

**Published:** 2018-01-08

**Authors:** Suriyati Mohamad, Nur Najihah Ismail, Thaigarajan Parumasivam, Pazilah Ibrahim, Hasnah Osman, Habibah A. Wahab

**Affiliations:** 1 School of Biological Sciences, Universiti Sains Malaysia, 11800 Minden, Pulau Pinang Malaysia; 2 School of Pharmaceutical Sciences, Universiti Sains Malaysia, 11800 Minden, Pulau Pinang Malaysia; 3 School of Chemical Sciences, Universiti Sains Malaysia, 11800 Minden, Pulau Pinang Malaysia

**Keywords:** *Costus speciosus*, *Cymbopogon citratus*, *Tabernaemontana coronaria*, Antituberculosis activity, Phytochemical identification, Growth kinetics, Cellular integrity

## Abstract

**Background:**

*Costus speciosus*, *Cymbopogon citratus*, and *Tabernaemontana coronaria* are herbal plants traditionally used as remedies for symptoms of tuberculosis (TB) including cough. The aims of the present study were to evaluate the in vitro anti-TB activity of different solvent partitions of these plants, to identify the phytochemical compounds, and to assess the effects of the most active partitions on the growth kinetics and cellular integrity of the tubercle organism.

**Methods:**

The in vitro anti-TB activity of different solvent partitions of the plant materials was determined against *M. tuberculosis* H37Rv using a tetrazolium colorimetric microdilution assay. The phytochemical compounds in the most active partition of each plant were identified using gas chromatography-mass spectrometry (GC-MS) analysis. The effects of these partitions on the growth kinetics of the mycobacteria were evaluated over 7-day treatment period in a batch culture system. Their effects on the mycobacterial cellular integrity were observed under a scanning electron microscope (SEM).

**Results:**

The respective n-hexane partition of *C. speciosus*, *C. citratus*, and *T. coronaria* exhibited the highest anti-TB activity with minimum inhibitory concentrations (MICs) of 100–200 μg/mL and minimum bactericidal concentration (MBC) of 200 μg/mL. GC-MS phytochemical analysis of these active partitions revealed that majority of the identified compounds belonged to lipophilic fatty acid groups. The active partitions of *C. speciosus* and *T. coronaria* exhibited high cidal activity in relation to time, killing more than 99% of the cell population. SEM observations showed that these active plant partitions caused multiple structural changes indicating massive cellular damages.

**Conclusions:**

The n-hexane partition of the plant materials exhibited promising in vitro anti-TB activity against *M. tuberculosis* H37Rv. Their anti-TB activity was supported by their destructive effects on the integrity of the mycobacterial cellular structure.

## Background

The scourge of *M. tuberculosis,* the etiologic agent of tuberculosis (TB) causes one of the world’s most devastating global health crises [1]. In 2015, the World Health Organisation (WHO) estimated that about 10.4 million people contracted TB and 1.4 million people died from this disease worldwide [2]. Even though TB mortality rate fell by 22% between 2000 and 2015, the inexorable threats arising from resistant and persistent TB are of major concern [2, 3]. With the limited presence of new anti-TB drugs, the global burden of TB continues to loom with an enormous toll in morbidity and mortality [4]. Therefore, the most urgent goal of TB chemotherapy is to develop highly active and low-cost drugs, which are readily produced [5, 6].

Among the possibilities to discover for potential new drugs are to prospect for these agents from natural resources such as plants. The primary advantages of using plants are that, they are inexhaustible sources of natural drugs as they provide a wealth of small molecules with drug-like properties and incredible structural diversity [7]. In addition, the profound therapeutic benefits of plant-derived medicines can mitigate many of the side effects that are often associated with synthetic drugs [8]. Ideally, the natural new drugs should also be more affordable and exhibit high bactericidal capacity. A high bactericidal capacity will result in a rapid decrease in the mycobacterial load and renders the patients less infectious [9].

As part of a continuous research to discover new potent and cheaper anti-TB agents from Malaysian ethnobotanical plants, we investigated the anti-TB potential of *Costus speciosus* (J. Koenig) Sm., *Cymbopogon citratus* (DC.) Stapf., and *Tabernaemontana coronaria* (Jacq.) Willd. since they are traditionally used as remedies to cure various respiratory diseases including cough [10–12]. These three plants are widely distributed in tropical countries and are commonly cultivated as garden herbs for use in cooking and for medicinal purposes. *C. speciosus* is an ornamental herb, which belongs to the family Coastaceae. Its common name is Malay or crepe ginger and its local name is setawar halia. *C. citratus* is a famous culinary herb, which is a member of the family Gramineae. Its common name is lemon grass and its local name is serai. The synonym of *T. coronaria* is *T. divaricata* (L.) R.Br. ex Roem. & Schult and it belongs to the family Apocynaceae. Its common name is crepe jasmine and its local name is akar susun kelapa. Many publications have reviewed and reported a variety of pharmacological properties of these medicinal plants including antibacterial, antifungal, anticough, anti-oxidant, antihelminthic, and antipneumonia [13–15]. In a previous preliminary study, we reported that the methanol extracts of *C. speciosus* and *T. coronaria* exhibited promising anti-TB activity with minimum inhibitory concentration (MIC) of 800 μg/mL against *M. tuberculosis* H37Rv, supporting their traditional uses in the treatment of TB [16]. In the present study, we investigated the in vitro anti-TB activity of different partitions of *C. speciosus*, *C. citratus*, and *T. coronaria* with emphasis on their effects on the growth kinetics and cellular integrity of the tubercle organism.

## Methods

### Collection and identification of plant materials

The plant materials were collected from identified areas in the northern states of Pulau Pinang and Kedah, West Malaysia. The plant species were authenticated by the Herbarium Unit, School of Biological Sciences, Universiti Sains Malaysia, where voucher specimens were deposited with designated numbers 11,435, 11,152, and 11,429 for *C. speciosus*, *C. citratus*, and *T. coronaria*, respectively. The plant materials (stems and flowers for *C. speciosus*, stems and rhizomes for *C. citratus*, and leaves for *T. coronaria*) were cleaned, dried in an oven at 40 °C, and pulverised into minute pieces.

### Partition preparation

Initial extraction of the pulverised dried plant materials was carried out by exhaustive maceration in 80% methanol for two to seven days at ambient temperature (26–27 °C). The crude methanol extracts were filtered (Whatman filter paper No. 1, England), and partially concentrated under reduced pressure at 40 °C using a rotary evaporator (Eyela, Japan). Then, the methanol extracts were sequentially partitioned using five solvents of different polarities: n-hexane, chloroform, ethyl acetate, and n-butanol (Merck, Germany). The partitioning process was carried out following the procedures as described previously [17] with minor modifications. Initially, 10–15 g of each crude methanol extract was dissolved completely in 200 mL mixture of methanol and distilled water (3:17, *v*/v) to produce an aqueous crude methanol extract. Then, an equal volume (200 mL) of n-hexane was added into this solution and shaken until well mixed. The mixture was poured into a separation funnel and swirled gently to ensure complete mixture. The separation funnel stopper was removed for a while to relieve the vapour pressure produced from mixing of the organic solvents. The n-hexane phase was collected when two distinct layers were observed and the solvent was changed. This process was repeated three times. During each change, the suspension of aqueous crude methanol extract was topped-up with distilled water to maintain the volume of 200 mL before new n-hexane solvent was added. The pooled n-hexane partition was dried completely in an oven at 40 °C, weighed, and stored at 4 °C prior to usage. The successive partitioning process was continued by replacing n-hexane with chloroform followed by ethyl acetate and n-butanol, consecutively. The final remaining extract from the aqueous crude methanol extract was labelled as aqueous partition. Stock solutions of the partitions were prepared at a concentration of 40 mg/mL in 100% dimethyl sulphoxide (DMSO). Prior to bioassay, working solutions of the partitions were prepared at a concentration of 3200 μg/mL (8% DMSO, *v*/v) by diluting the stock solutions in sterile distilled water. The working solutions were then sterilised by filtration using a cellulose membrane (Sartorius, Germany) of 0.22 μm pore size.

### Preparation of *Mycobacterium tuberculosis* H37Rv inoculum

The test organism, *M. tuberculosis* H37Rv ATCC 25618 was grown on Middlebrook (MB) 7H10 agar (Difco, USA) enriched with oleic acid, albumin, dextrose, and catalase (OADC) (Difco, USA) for 10 days at 37 °C in 8% CO_2_. The inoculum for the determinations of MIC and minimum bactericidal concentration (MBC) was prepared by suspending the 10-day old mycobacterial cells in 10 mL of MB7H9 broth (Difco, USA) enriched with albumin, dextrose, and catalase (ADC) (Difco, USA) and incubated for three days to attain log phase of growth. The turbidity of the cell culture was first adjusted to McFarland standard No. 1 (equivalent to approximately 3 × 10^7^ colony forming units (CFU)/mL) and then was further diluted 1:20 in MB7H9-OADC broth to give a final inoculum concentration of approximately 1.5 × 10^7^ CFU/mL. A different inoculum was prepared for the growth kinetics study by suspending the 10-day old mycobacterial cells grown on MB7H10 agar in 10 mL of MB7H9-ADC broth and the turbidity of the cell suspension was immediately adjusted to McFarland standard No. 3, equivalent to a density of approximately 9 × 10^8^ CFU/mL. All procedures involving handling of the organism were carried out under very strict containment conditions in class 2^+^ biosafety cabinets to eliminate biohazards.

### Determination of minimum inhibitory concentration and minimum bactericidal concentration values of plant solvent partitions against *Mycobacterium tuberculosis* H37Rv

The determination of MIC value of the plant solvent partitions against *M. tuberculosis* H37Rv were performed using a colorimetric tetrazolium microdilution assay based on the technique as described previously [18] and modified slightly as in our earlier report [16]. Each partition was tested in triplicates at least twice until reproducible results were consistently obtained. Briefly, 200 μL of sterile distilled water was added into all outer perimeter wells of two sets of sterile 96-well microtitre plates (TPP, Germany) in duplicate to prevent dehydration during incubation. The microplates of Set 1 were for the determination of MIC and Set 2 were for the determination of MBC. Then, 100 μL of MB7H9-OADC broth was added into all wells except the first test column of each microplate. A volume of 100 μL of each partition working solution was then added into the wells in the first and second test columns in triplicates. By using a multi-channel pipette, a two-fold dilution series of the partition was made by initially transferring 100 μL from the wells in the second to the third test columns. Control wells containing anti-TB drug, isoniazid and untreated control wells with no partition or drug were included in each microplate. Next, 100 μL of *M. tuberculosis* H37Rv inoculum was added into all wells. The test concentrations of the plant partitions ranged from 1600 to 50 μg/mL. The test concentrations of isoniazid ranged from 1.25 to 0.039 μg/mL. The final concentration of DMSO in the highest test concentration of partitions was 4% *v*/v. Control experiments showed that 4% DMSO did not inhibit the growth of the mycobacterial cells. The microplates were sealed and incubated at 37 °C in 8% CO_2_ for five days. On the 5th day, 50 μL of freshly prepared tetrazolium reagent mixture (1:1, v/v) of 3-(4,5-dimethylthiazol-2-yl)-2,5-diphenyl-tetrazolium bromide (MTT) (Sigma, USA) at a dilution of 1 mg/mL in absolute ethanol and sterile 10% Tween 80 was added into the wells of microplates Set 1 and reincubated for another 24 h. The results were read visually on the 6th day, whereby; a colour change from yellow to purple indicated growth. In living cells, MTT is reduced to an insoluble purple formazan by the mitochondrial dehydrogenases and reductases of the cells associated with the regulation of cell growth, proliferation, and differentiation [19, 20]. Hence, the MIC was defined as the lowest partition concentration, which prevented the colour change of tetrazolium reagent from yellow to purple, at which visible growth was inhibited.

The determination of MBC value of the plant solvent partitions was performed based on the technique as described by the Clinical and Laboratory Standards Institute (CLSI) [21] with appropriate modifications. In continuation of the MIC determination, on the 6th day, a loopful of the culture broth from each test well in microplates Set 2 corresponding to the yellow well (no growth) in microplates Set 1 was streaked onto MB7H10-OADC agar plate in duplicates. The plates were sealed and incubated for 21 days. The CFU were observed and the MBC was defined as the lowest partition concentration, which prevented total growth of the mycobacteria, at which 99.9% of the final inoculum was killed.

### Identification of phytochemical compounds in active n-hexane partitions

As GC-MS technique is an ideal technique for the characterisation of volatile and semi-volatile organic compounds of plant origins in non-polar plant extracts [22], it was used to identify the compounds contained in the active n-hexane partition in this study. Besides, it is concise, efficient, and the automated system gives fast, reproducible, and effective results. The GC-MS analysis was performed at the National Poison Centre, Universiti Sains Malaysia following a standard operational protocol. Briefly, 3 mg of the dried n-hexane partition of each plant was dissolved in 0.1 mL of n-hexane. One microlitre of each mixture was injected into the GC-MS system for the requisite analysis. The system consisted of a Hewlett Packard HP7890 GC (California, USA), an auto sampler, HP5979 mass selective detector, and MSD ChemStation (E.02.01.1177) software. The column was an HP-5MS fused silica capillary column (30 m × 0.25 mm i.d) with cross-linked 5% phenyl methyl siloxane (0.25 μm film thickness). The carrier gas was helium at 2.1 mL/min. The oven temperature was 50 °C and held for five minutes. The temperature was increased linearly at 25 °C per minute to 300 °C. The final temperature was held for 10 min. The inlet and detector temperatures were both set at 280 °C. Full scan mode was performed using electron ionisation at 70 eV. The total run time was 25 min. The identification of the compounds was based on mass spectral matching (>80%) with reference standards in the National Institute of Standards and Technology library (NIST Version 2.0, Oct 22, 2009) and by direct comparison with published data. Then the name, molecular weight, and structure of the compounds of the partitions were ascertained.

### Evaluating the effects of active n-hexane partitions on the growth kinetics of *Mycobacterium tuberculosis* H37Rv

The active n-hexane partitions of *C. speciosus* (MIC: 100 μg/mL)*, C. citratus* (MIC:200 μg/mL)*,* and *T. coronaria* (MIC: 100 μg/mL) were further evaluated to observe their time-dependent effects on the growth kinetics of *M. tuberculosis* H37Rv using the growth study procedure in a batch culture system as described previously [23]. Briefly, 2 mL of the mycobacterial inoculum (approximate concentration of 9 × 10^8^ CFU/mL) was inoculated into five sets of culture bottles in duplicates containing 18 mL of MB7H9-OADC broth and 20 sterile glass beads (3 mm diameter) for easy mixture. The first three sets of culture bottles (Set 1 - Set 3) contained different test partitions at a final concentration of their respective MIC values. Set 4 contained control drug, isoniazid at its MIC of 0.078 μg/mL, whereas, Set 5 served as a positive growth control without partition or drug. The culture bottles were incubated at 37 °C in 8% CO_2_ for seven days with gentle occasional swirling to prevent cellular aggregations, which could result in inaccurate counts. Samples were taken daily from the culture bottles for the colony counts using a modification of the Miles and Misra drop plate method [24]. Briefly, ten-fold serial dilutions of the culture samples were prepared in phosphate buffer saline (PBS) solution. A volume of 20 μL of each diluent was dropped in triplicates onto two sets of MB7H10-OADC agar plates. The plates were allowed to dry at room temperature (26–27 °C), sealed and incubated at 37 °C in 8% CO_2_ for 21 days. After incubation, the colonies were counted and the CFU per mL were calculated.

### Observing the effects of active n-hexane partitions on the cellular integrity of *Mycobacterium tuberculosis* H37Rv

The effects of n-hexane partitions of *C. speciosus, C. citratus,* and *T. coronaria* at their respective MIC values on the integrity of the mycobacterial cells were observed under a scanning electron microscope (SEM). The cells were harvested from Day 3 of the study on the growth kinetics of *M. tuberculosis* H37Rv. This was so, because on Day 3, the effects on the cellular integrity could be observed on both viable and dead cells, based on the results obtained for the growth kinetics study. The Electron Microscope Unit, School of Biological Sciences, Universiti Sains Malaysia performed sample preparation for SEM observation following a standard protocol. Briefly, each sample of the treated mycobacterial cells in 7H9-OADC broth was centrifuged at 1850 g for five minutes. The supernatant was discarded and the pellet was resuspended in McDowell-Trump fixative solution prepared in 0.1 M PBS for at least two hours. The sample suspension was then centrifuged, supernatant discarded, and the pellet was washed with 0.1 M PBS twice and post-fixed in osmium tetroxide prepared in PBS for one hour. The sample was again centrifuged and washed with distilled water twice. The pellet was dehydrated through a series of 50%, 75%, 95%, and 100% ethanol followed by hexamethyldisilazane (HMDS) for 10 min each. The pellet was centrifuged and resuspended after each ethanol change. Lastly, after HMDS dehydration, the pellet was centrifuged and HMDS was decanted. The vials containing the cells were placed in a desiccator to air-dry at room temperature (26–27 °C). The dried cells were mounted onto a SEM specimen stub with a double-sided sticky tape and coated with gold. Then the cells were observed under Leo Supra 50VP Field Emission SEM equipped with Oxford INCA 400 energy dispersive X-ray microanalysis system (Carl-Zeiss, Germany). The effects of the plant partitions on the cellular integrity of *M. tuberculosis* were compared to the effects of the isoniazid-treated and untreated control cells.

## Results

### Minimum inhibitory concentration and minimum bactericidal concentration of plant solvent partitions against *Mycobacterium tuberculosis* H37Rv

The TB inhibitory and bactericidal activities of the plant partitions based on MIC and MBC values are shown in Table 1.

**Table 1:** Minimum inhibitory concentration and minimum bactericidal concentration values of the plant solvent partitions against *Mycobacterium tuberculosis* H37Rv

Plants	Local names/Traditional uses	Herbarium vouchers	Plant parts	Plant partitions and MIC and MBC (μg/mL)											
				Methanol		n-Hexane		Chloroform		Ethyl acetate		n-Butanol		Aqueous	
				MIC	MBC	MIC	MBC	MIC	MBC	MIC	MBC	MIC	MBC	MIC	MBC
*Costus speciosus* (J. Koenig) Sm. (Costaceae)	Setawar halia/Cough	11435	Stem-flower	800	ND	100	200	200	200	400	ND	1600	ND	1600	ND
*Cymbopogon citratus* (DC. ex Nees) Stapf. (Graminae)	Serai/Cough	11152	Stem-rhizome	NI	ND	200	NC	1600	ND	1600	ND	NI	ND	NI	ND
*Tabernaemontana coronaria* (L.) Willd. (Apocynaceae)	Akar susun kelapa/Cough	11429	Leaf	800	ND	100	200	800	ND	800	ND	400	ND	1600	ND

“NI”: not showing inhibition even at the highest test concentration of 1600 μg/mL. “NC”: not showing cidal effect even at the highest test concentration of 1600 μg/mL. “ND”: MBC not determined. All positive control wells showed bacterial growth. Negative growth control drug, isoniazid exhibited consistent MIC and MBC values of 0.078 μg/mL

In this study, we interpreted positive anti-TB activity as any values of MIC ≤1600 μg/mL, which was the highest test concentration. MBC was only determined for partitions with MIC ≤200 μg/mL (based on the general values of our unpublished data). As indicated by the results in Table 1, all the stem-flower partitions of *C. speciosus* exhibited varying anti-TB activity with MICs of 1600–100 μg/mL. The highest activity was exhibited by n-hexane partition (MIC: 100 μg/mL; MBC: 200 μg/mL), followed by chloroform partition (MIC: 200 μg/mL; MBC: 200 μg/mL). The stem-rhizome partitions of *C. citratus* that exhibited anti-TB activity were n-hexane (MIC: 200 μg/mL), chloroform, and ethyl acetate (MIC: 1600 μg/mL). MBC was determined for its n-hexane partition but there was no cidal effect shown even at the highest test concentration of 1600 μg/mL. All the leaf partitions of *T. coronaria* exhibited anti-TB activity with MICs of 100–1600 μg/mL. The highest activity was also exhibited by n-hexane partition (MIC: 100 μg/mL; MBC: 200 μg/mL). The polar partitions of methanol, n-butanol and aqueous of these three plants exhibited poor or no activity against the mycobacterial cells. The untreated control wells consistently showed growth of the mycobacterial cells and the control drug, isoniazid exhibited consistent inhibition with MIC of 0.078 μg/mL.

### Identification of phytochemical compounds in active n-hexane partitions

**Fig. 1:**
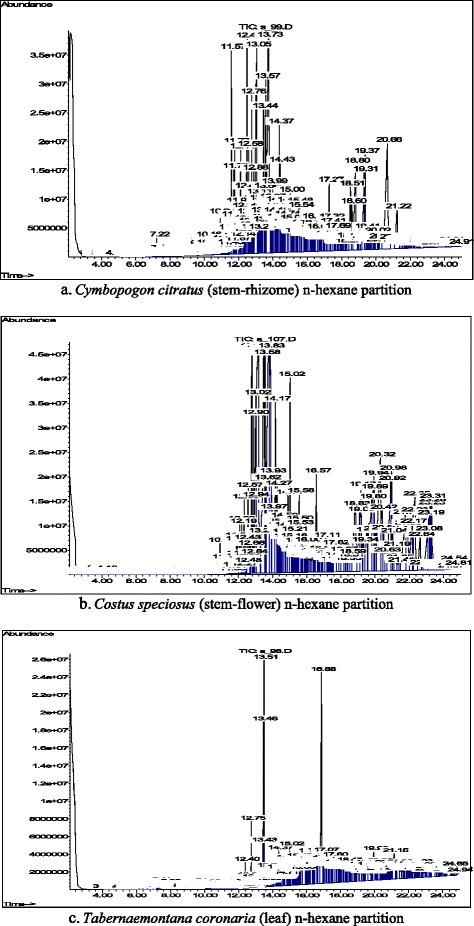
**a**-**c** Gas chromatography-mass spectrometry chemometric profiles of plant n-hexane partitions

**Fig. 2:**
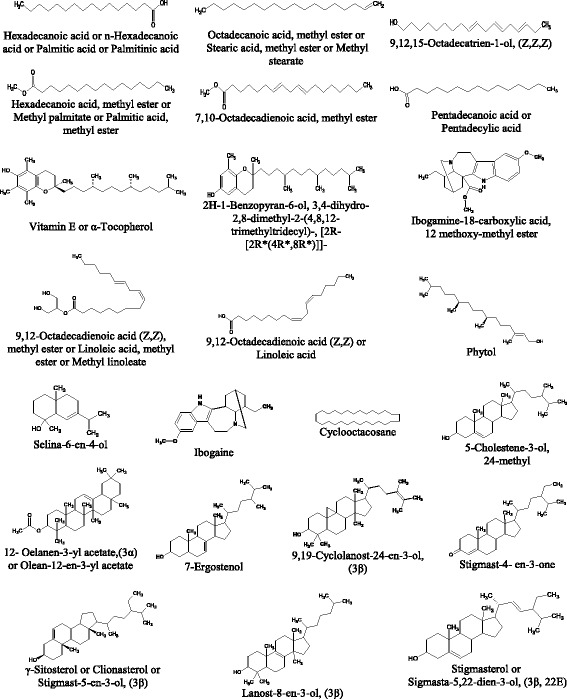
Molecular structures of major compounds from each plant n-hexane partition based on gas chromatography-mass spectrometry chemometric profiling

In this study, preliminary phytochemical analysis was carried out to identify the compounds that could be present in the most active partition of each plant. The compounds were characterised and identified by comparison of their mass fragmentation patterns with NIST database library and published data. Figure 1a–c show the GC-MS chemometric profiles of n-hexane partition of *C. speciosus, C. citratus*, and *T. coronaria*, respectively. In general, each plant n-hexane partition revealed a mixture of large number of phytochemicals that constituted of almost all volatile and semi-volatile organic compounds. These compounds represented different major phytochemical classes including sterol, phenol, diterpene, sesquiterpene, alkaloid, triterpenoid, fatty acid, and tocopherol. Table 2a–c show the major compounds identified in each n-hexane partition of *C. speciosus, C. citratus*, and *T. coronaria*, respectively. Their molecular structures are shown in Fig. 2.

**Table 2:** a-c Major phytochemical compounds identified in the active plant n-hexane partitions by gas chromatography-mass spectrometry chemometric profiling

No.	Retention time (min)	Peak area	% of total peak area	Name of compound	Library match (%)	NIST No.	CAS No.	Molecular formula	Molecular weight
a *Costus speciosus* (stem-flower) n-hexane partition^a^									
1	12.94	2.150.E + 08	7.37	Hexadecanoic acid or n-Hexadecanoic acid or Palmitic acid or Palmitinic acid	95	151,973	57–10-3	C_16_H_32_O_2_	256
2	13.514	3.480.E + 09	7.25	9,12-Octadecadienoic acid (Z,Z), methyl ester or Linoleic acid, methyl ester or Methyl linoleate	99	233,849	112–63-0	C_19_H_34_O_2_	294
3	12.793	1.560.E + 09	2.68	Hexadecanoic acid, methyl ester or Methyl palmitate or Palmitic acid, methyl ester	99	158,970	112–39-0	C_17_H_34_O_2_	270
4	13.577	9.940.E + 08	1.71	Octadecanoic acid, methyl ester or Stearic acid, methyl ester or Methyl stearate	98	79,123	112–61-8	C_19_H_38_O_2_	298
5	19.165	9.070.E + 08	1.56	Lanost-8-en-3-ol, (3β)	91	253,595	79–62-9	C_30_H_52_O	428
6	20.985	8.240.E + 08	1.42	Stigmast-4-en-3-one	95	17,165	1058–61-3	C_29_H_48_O	412
7	12.569	6.730.E + 08	1.16	Pentadecanoic acid or Pentadecylic acid	99	63,741	1002–84-2	C_15_H_30_O_2_	242
8	19.095	6.670.E + 08	1.15	7-Ergostenol	86	253,005	116,179–22-7	C_28_H_48_O	400
9	16.567	5.880.E + 08	1.01	2H-1-Benzopyran-6-ol, 3,4-dihydro-2,8-dimethyl-2-(4,8,12-trimethyltridecyl)-, [2R-[2R*(4R*,8R*)]]-	98	151,380	119–13-1	C_27_H_46_O_2_	402
b *Cymbopogon citratus* (stem-rhizome) n-hexane partition^b^									
1	13.731	2.852.E + 08	9.16	9,12-Octadecadienoic acid (Z,Z) or Linoleic acid	93	229,327	60–33-3	C_18_H_32_O_2_	280
2	18.808	8.335.E + 08	2.68	Stigmasterol or Sigmasta-5,22-dien-3-ol, (3β, 22E)	90	251,057	83–48-7	C_29_H_48_O	412
3	13.444	6.416.E + 08	2.06	7,10-Octadecadienoic acid, methyl ester	95	35,764	56,554–24-6	C_19_H_34_O_2_	294
4	19.368	5.734.E + 08	1.84	γ-Sitosterol or Clionasterol or Stigmast-5-en-3-ol, (3β)	99	151,558	83–47-6	C_29_H_50_O	414
5	11.575	7.538.E + 08	1.73	Selina-6-en-4-ol	94	140,232	NA	C_15_H_26_O	222
6	18.514	4.958.E + 08	1.59	5-Cholestene-3-ol, 24-methyl	99	214,174	NA	C_28_H_48_O	400
7	17.274	3.632.E + 08	1.17	Cyclooctacosane	95	72,382	297–24-5	C_28_H_56_	392
8	12.758	9.362.E + 08	1.16	Hexadecanoic acid, methyl ester or Methyl palmitate or Palmitic acid, methyl ester	98	158,970	112–39-0	C_17_H_34_O_2_	270
c *Tabernaemontana coronaria* (leaf) n-hexane partition^c^									
1	16.882	7.578.E + 08	6.91	Ibogamine-18-carboxylic acid, 12 methoxy-methyl ester	96	48,516	510–22-5	C_22_H_28_N_2_O_3_	368
2	13.507	3.355.E + 08	3.06	Phytol	91	14,111	150–86-7	C_20_H_40_O	296
3	13.458	2.298.E + 08	2.10	9,12,15-Octadecatrien-1-ol, (Z,Z,Z)	93	141,196	506–44-5	C_18_H_32_O	264
4	21.146	1.781.E + 08	1.63	12-Oelanen-3-yl acetate, (3α) or Olean-12-en-3-yl acetate	95	244,056	33,055–28-6	C_32_H_52_O_2_	468
5	16.693	1.786.E + 08	1.63	Ibogaine	99	131,027	83–74-9	C_20_H_26_N_2_O	310
6	17.596	1.501.E + 08	1.37	Vitamin E or α-Tocopherol	98	151,382	59–02-9	C_29_H_50_O_2_	430
7	16.532	1.482.E + 08	1.35	2H-1-Benzopyran-6-ol, 3,4-dihydro-2,8-dimethyl-2-(4,8,12-trimethyltridecyl)-, [2R-[2R*(4R*,8R*)]]-	95	151,380	119–13-1	C_27_H_46_O_2_	402
8	19.956	1.231.E + 08	1.12	9,19-Cyclolanost-24-en-3-ol, (3β)	98	68,266	25,692–13-1	C_31_H_52_O	440

^a^Number of compounds detected: 261; Number of compounds identified: 54

^b^NA: Not available; Number of compounds detected: 308; Number of compounds identified: 36

^c^Number of compounds detected: 323; Number of compounds identified: 31

The GC-MS chemometric profile of *C. speciosus* stem-flower n-hexane partition (Fig. 1a) revealed the presence of 261 different compounds, of which 54 were identified. Amongst the 54 identified compounds, hexadecanoic acid or palmitic acid or palmitinic acid; 9,12-octadecadienoic acid (Z,Z), methyl ester or methyl linoleate; hexadecanoic acid, methyl ester or palmitic acid, methyl ester; octadecanoic acid, methyl ester; lanost-8-en-3-ol (3β); stigmast-4-en-3-one; pentadecanoic acid or pentadecylic acid; 7-ergostenol; and 2H-1-benzopyran-6-ol, 3,4-dihydro-2,8-dimethyl-2-(4,8,12-trimethyltridecyl-2R-2) constituted the major compounds in this partition (Table 2a, Fig. 2). The other 45 identified compounds were found in trace amounts of ≤1.0% of the total peak area. The GC-MS chemometric profile of *C. citratus* stem-rhizome n-hexane partition (Fig. 1b) revealed the presence of 308 different compounds, of which 36 were identified. The eight major compounds with ≥1.0% of the total peak area were 9,12-octadecadienoic acid (Z,Z); stigmasterol; 7,10-octadecadienoic acid, methyl ester; γ-sitosterol; selina-6-en-4-ol; 5-cholestene-3-ol, 24-methyl; cyclooctacosane; and hexadecanoic acid, methyl ester (Table 2b, Fig. 2). Lastly, the GC-MS chemometric profile of *T. coronaria* leaf n-hexane partition (Fig. 1c) revealed the presence of 323 different compounds, of which 31 were identified. Of these 31 compounds, ibogamine-18-carboxylic acid, 12-methoxy, methyl ester; phytol; 9,12,15-octadecatrien-1-ol (Z,Z,Z); 12-oleanen-3-yl acetate, (3α); ibogaine; vitamin E; 2H-1-benzopyran-6-ol, 3,4-dihydro-2,8-dimethyl-2-(4,8,12-trimethyltridecyl)-, [2R-[2R*(4R*,8R*)]]-; and 9,19-cyclolanost-24-en-3-ol (3β) constituted the major compounds (Table 2c, Fig. 2). The remaining identified compounds were found in trace amounts of ≤1.0% of the total peak area.

### Effects of n-hexane plant partitions on the growth kinetics of *Mycobacterium tuberculosis* H37Rv

The results of this study were expressed as the number of colonies per mL or CFU/mL of *M. tuberculosis* H37Rv over 7-day treatment period as shown in Table 3.

**Table 3:** Effects of n-hexane plant partitions on the colony counts of *Mycobacterium tuberculosis* H37Rv over 7-day treatment period

Days	Control positive (no drug/extract)			Isoniazid (MIC: 0.078 μg/mL)			*C. speciosus* (MIC: 100 μg/mL)			*C. citratus* (MIC: 200 μg/mL)			*T. coronaria* (MIC: 100 μg/mL)		
	Mean CFU/mL	Mean % of day 0	Mean % STDEV	Mean CFU/mL	Mean % of day 0	Mean % STDEV	Mean CFU/mL	Mean % of day 0	Mean % STDEV	Mean CFU/mL	Mean % of day 0	Mean % STDEV	Mean CFU/mL	Mean % of day 0	Mean % STDEV
0	1.36 _x_ 10^6^	100.00	0.87	1.29 _x_ 10^6^	100.00	4.56	1.86 _x_ 10^6^	100.00	0.32	9.00 _x_ 10^6^	100.00	5.24	4.92 _x_ 10^6^	100.00	7.19
1	1.48 _x_ 10^6^	108.59	5.59	1.04 _x_ 10^6^	80.65	1.13	8.08 _x_ 10^5^	43.60	4.37	3.58 _x_ 10^6^	39.81	9.87	8.42 _x_ 10^5^	17.12	4.20
2	2.27 _x_ 10^6^	166.87	4.16	1.80 _x_ 10^5^	13.87	9.87	7.25 _x_ 10^5^	39.10	8.13	1.13 _x_ 10^6^	12.59	6.24	6.17 _x_ 10^5^	12.54	4.59
3	2.50 _x_ 10^6^	184.05	0.00	1.58 _x_ 10^5^	12.26	4.47	1.35 _x_ 10^5^	7.28	5.24	9.42 _x_ 10^5^	10.46	1.25	5.08 _x_ 10^5^	10.34	11.59
4	2.71 _x_ 10^6^	199.39	2.18	9.92 _x_ 10^4^	7.68	5.94	1.07 _x_ 10^5^	5.75	8.84	8.75 _x_ 10^5^	9.72	6.73	8.08 _x_ 10^4^	1.64	1.46
5	3.58 _x_ 10^6^	263.80	3.29	7.92 _x_ 10^4^	6.13	1.49	1.67 _x_ 10^2^	0.01	0.00	5.92 _x_ 10^5^	6.57	5.98	7.83 _x_ 10^4^	1.59	6.02
6	4.50 _x_ 10^6^	331.29	5.24	6.58 _x_ 10^4^	5.10	5.37	1.67 _x_ 10^2^	0.01	0.00	5.92 _x_ 10^5^	6.57	1.99	4.25 _x_ 10^4^	0.86	2.77
7	3.08 _x_ 10^6^	226.99	3.82	3.67 _x_ 10^4^	2.84	12.86	1.67 _x_ 10^2^	0.01	0.00	3.83 _x_ 10^5^	4.26	0.00	4.25 _x_ 10^4^	0.86	2.77

Note: The mean CFU/mL was calculated from two sets of CFU/mL values in triplicate and the mean percentage of standard deviation (STDEV) was calculated based on the differences between two sets of percentage CFU/mL of Day 0

**Fig. 3:**
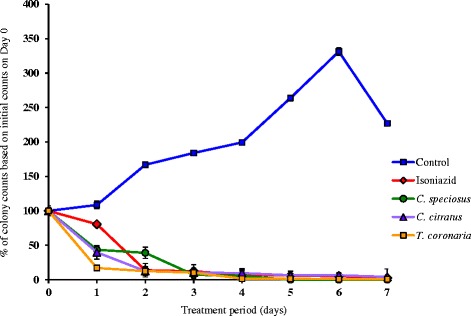
Effects of n-hexane plant partitions on the growth kinetics of *Mycobacterium tuberculosis* H37Rv over 7-day treatment period

These values were extrapolated as the percentage of colony counts based on the initial counts on Day 0 to show the effects of the partitions on the mycobacterial growth kinetics as illustrated in Fig. 3 and summarised in Table 4.

**Table 4:** Overall key effects of n-hexane plant partitions on the growth kinetics of *Mycobacterium tuberculosis* H37Rv

Drug/Plant sp.	MIC (μg/mL)	Evidence of cidal activity (≥ 90% killing)	Cidal activity (≥ 99% killing)	Highest killing rate achieved (%)	Bacteriostatic/bactericidal
Isoniazid	0.078	Day 4	Not achieved	97.16	Bacteriostatic
*C. speciosus*	100	Day 3	Day 5	99.99	Bactericidal
*C. citratus*	200	Day 4	Not achieved	95.74	Bacteriostatic
*T. coronaria*	100	Day 3	Day 6	99.14	Bactericidal

The results were compared to the negative and positive growth controls. To date, there are no specific guidelines available to rate the cidal activity of mycobacteria and the interpretive criteria of bactericidal activity of antimycobacterial agents are not uniform. In the present study, the results were interpreted based on the definition of cidal or sterilising activity as an effective reduction of the bacterial counts by ≥99% [25–27] and evidence of bactericidal activity as a reduction of colony counts by ≥90% (decrease to 10% or below of initial count) [28]. Accordingly, in this study, we interpreted final killing effect of ≥90% but <99% in the reduction of colony counts as bacteriostatic activity. Figure 3 shows that the untreated control mycobacterial cells underwent three growth phases when introduced into a new culture medium in the absence of anti-TB agents. The growth phases comprised of a lag phase from Day 0 to Day 1, a log phase of gradual increase in colony counts from Day 1 to Day 4, an exponential increase from Day 4 to Day 6, and a death phase after Day 6. On the other hand, the control cells treated with isoniazid at its MIC of 0.078 μg/mL showed that on Day 1, the colony counts were decreased by 19.35% and the time-kill effect of the drug was apparent with a sharp decline by about 86% of the colony counts on Day 2 (Table 4). On Day 4, the colony counts decreased by 92.32%, showing evidence of mycobactericidal activity (≥ 90% killing). By the end of the study period, a mycobacteriostatic activity of 97.16% killing was obtained but sterilising activity (≥ 99% killing) was not achieved. Essentially, the effects of stem-rhizome n-hexane partition of *C. citratus* on the growth kinetics of *M. tuberculosis* emulated that of isoniazid (Fig. 3). Similarly, evidence of mycobactericidal activity was observed on Day 4 by a reduction of 90.28% in the colony counts and sterilising activity was not achieved during the study period (Table 4). This partition attained mycobacteriostatic activity of 95.74% killing on Day 7. During the first two days of exposure to *C. speciosus*, the mycobacterial cell population decreased by about 60% (Fig. 3 and Table 4). Thereafter, exponential bactericidal activity was observed on Day 3 with a sharp decrease in colony counts by 92.72%. By Day 5, almost all cells were killed (99.99% killing). Whereas, exposure to *T. coronaria* resulted in immediate exponential bactericidal activity with a sharp decrease of the cell population by 82.88% on Day 1 (Fig. 3 and Table 4). Evidence of mycobactericidal activity was achieved on Day 3 as shown by a reduction of about 90% in the colony counts. By Day 6, sterilising activity was achieved (99.14% killing).

### Effects of n-hexane plant partitions on the cellular integrity of *Mycobacterium tuberculosis* H37Rv

**Fig. 4:**
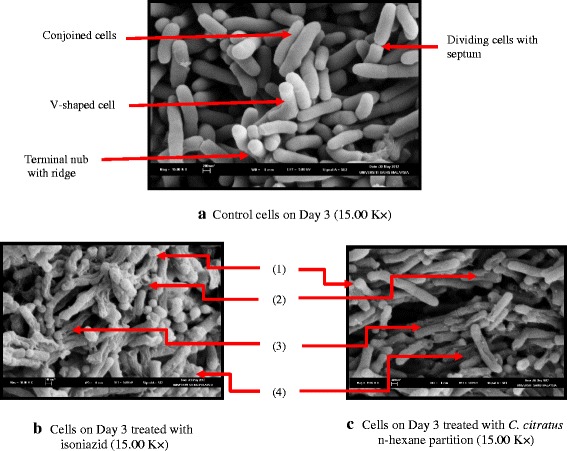
**a**-**c** Scanning electron microscopy observation of the cellular integrity of *Mycobacterium tuberculosis* H37Rv. (1) Bulges at cell poles, (2) Hollow shells, (3) Holes and dents on cell surface, (4) Amorphous mass of cell debris with web-like materials

A number of studies have demonstrated the destructive effects of antibiotics and chemical compounds on the mycobacterial cells, describing a variety of morphological defects [29–31]. In the present study, the effects of the active n-hexane partitions of *C. speciosus, C. citratus*, and *T. coronaria* at their MIC values on the cellular integrity of *M. tuberculosis* H37Rv were observed under SEM to provide physical evidence of cellular changes for the growth study results as described above. The effects of the plant partitions on the cells of *M. tuberculosis* were compared to the effects of the isoniazid-treated and untreated control cells as shown in Fig. 4a–c.

The control cells (Fig. 4a) on Day 3 was a mixture of numerous cells with smooth surface, well defined rigid shape of different morphologies, and some cells with wrinkled surface. Majority of the control cells were mostly rod-like bacilli with septa or constrictions and other types of cell shapes (curve, V or Y-shape) occurred in lower frequency. In addition, some cells were also observed to be conjoined parallel to each other and a few cells had terminal nubs with ridges. Figure 4b shows the effects of isoniazid treatment on the integrity of the mycobacterial cells. Figure 4c represents the effects of treatment with the three n-hexane plant partitions as the differences in their individual effects were not discernible. The effects of the plant partitions were generally similar to the isoniazid treatment. However, compared to the control untreated cells, the cells treated with the plant partitions and isoniazid showed significant cellular alterations. After three days of exposures to the partitions and the drug, the cell population consisted of a majority of degraded cells and very few viable cells with smooth surface and rigid outline remained (Fig. 4b and c). The degraded cells appeared wrinkled, ragged, and shrunk. Tiny holes and dents were also observed on the cell surfaces. Some cells looked like empty shells with hollow ends. Another feature in this study was the observation of patches of amorphous mass of cell debris with “web-like” materials. Slight alterations of the poles of some treated cells were also observed, a feature that was not observed with the control cells. These treated bacilli swelled slightly at either end, so club shape and bulges were observed.

## Discussion

All the plant partition samples were dried thoroughly in an oven at 40 °C to ensure that any remaining solvent residues evaporated completely. This important step was taken to eliminate the intrinsic toxic effect of the solvents on the test organism, which could interfere with the assay results. All the three test plants exhibited a variety of in vitro anti-TB activity against *M. tuberculosis* H37Rv. Based on their MIC and MBC values, the most active partitions were n-hexane partitions of *C. speciosus* (stem and flower) and *T. coronaria* (leaf)*,* with promising MIC of 100 μg/mL and MBC of 200 μg/mL. These results indicate that these partitions could contain the highest amount of bioactive constituents. The other partition that possessed good anti-TB activity with MIC of 200 μg/mL was *C. speciosus* chloroform and *C. citratus* n-hexane. Reviews on anti-TB activity of natural products showed that plant extracts with inhibitory concentrations of ≤100 μg/mL have a high potential of containing active phytochemical constituents [32, 33]. However, it is also possible that extracts with MICs of 100–200 μg/mL could contain promising compounds. Based on the types of solvent, n-hexane produced the highest anti-TB activity, followed by chloroform, and ethyl acetate, indicating that a higher amount of bioactive constituents against *M. tuberculosis* were possibly extracted by the non-polar solvents especially n-hexane. These results agreed with other anti-TB studies of natural products [34, 35]. Since non-polar solvents are used to solubilise mostly lipophilic compounds such as alkanes, fatty acids, sterols, some terpenoids, alkaloids, and coumarins [36], the results in this study indicate that most of the active constituents could be of lipophilic nature. These results were also supported by the poor activity observed for the polar partitions of methanol, n-butanol and aqueous. It was possible that the bioactive constituents in these partitions could be absent or their amounts were too little to exert inhibitory effects on the mycobacterial cells. Alternatively, since the majority of constituents in these polar solvent partitions were expected to comprise of hydrophilic groups, the molecules could not traverse the lipid layer of the mycobacterial outer membrane. The outer membrane of *M. tuberculosis* is known to present a barrier to the penetration of hydrophilic molecules due to the fact that the cell wall is double-layered, comprising of an inner electron-dense layer of peptidoglycan and an outer electron-transparent layer containing mycolyl arabinogalactan complex covalently bound to the peptidoglycan [37, 38]. In this study, the potent anti-TB activity of isoniazid (MIC and MBC: 0.078 μg/mL) was expected in selecting isoniazid as the control drug due its exquisite specificity to *M. tuberculosis* [39, 40]. It should be noted that the inclusion of this control drug in this study was not to gauge the activity of the plant partitions; rather, it served specifically as a negative growth control to validate the assay procedures together with the positive control.

Gas chromatography-mass spectrometry analysis of the n-hexane partition of each plant revealed a mixture of large number of phytochemicals, of which majority were volatile and semi-volatile organic compounds of lipophilic nature. It must be noted that the numbers of identified compounds and their identities were not absolute for each partition as the results presented in this study were limited and restricted as per the materials utilised and the GC-MS methods employed. The identified compounds, which represented phytochemical classes such as sterol, phenol, diterpene, sesquiterpene, alkaloid, triterpenoid, fatty acid, and tocopherol were also commonly isolated in antitubercular plants [41–43]. The lipophilic nature of the compounds was an important property of the bioactive constituents in these plant partitions as they could penetrate the hydrophobic outer membrane of *M. tuberculosis* to exert inhibitory effects. The identification of these compounds supported the higher anti-TB activity of the non-polar solvent partitions as discussed above. Ideally, the specific bioactive constituents should be isolated and elucidated. At present we are working with our research collaborators to further isolate and elucidate these constituents, which require an arduous lengthy chemical-biological approach. Nevertheless, with the recent availability of a number of modern sophisticated hyphenated separation and spectroscopic techniques, their isolation and elucidation should become much easier. With the isolation of these active constituents, further assays could be carried out to identify the mechanism of their actions on the tubercle cells.

The active n-hexane partitions of *C. speciosus, C. citratus*, and *T. coronaria* were further investigated to provide an insight into the killing dynamics of these partitions at their respective MIC values by assessing the mycobacterial killing at different time-points during their exposures. As exposure of the mycobacterial population to maximum achievable drug concentrations in vivo lasts for only a very short period [26], the in vitro endpoint assay approach of MIC and MBC determinations could not reflect the actual events during infection. Therefore, to emphasise the susceptibility results, inhibition rate of the mycobacterial growth was also assessed. Moreover, according to the recommendation by the National Committee for Clinical Laboratory Standards (NCCLS), a time factor should be included in antimicrobial susceptibility methods because the rate of killing has more clinical significance than the degree of killing [44]. Particularly, during *M. tuberculosis* initial infection, the antimicrobial killing rate in relation to the time of exposure is an important characteristic for therapeutic efficacy to prevent development of resistance [27]. In this study, the growth features of consecutive lag, log, and death phases exhibited by the untreated control mycobacterial cells were typical of the normal growth of *Mycobacterium* species when introduced into a new medium [27, 45, 46]. Concerning the treatment with isoniazid, even though sterilising activity (≥ 99% killing) was not achieved, less than 3% of the mycobacterial cell population remained viable by the end of the study period, indicating a strong mycobacteriostatic activity. This result concurred with previous findings that isoniazid is mycobacteriostatic against *M. tuberculosis* at lag or initial phase as opposed to log phase, during which, it is mycobactericidal [28, 47]. In this present study, it was expected that the mycobacterial cell population was still undergoing lag phase of growth since the starting inoculum was at lag phase. The mycobacteriostatic activity of stem-rhizome n-hexane partition of *C. citratus* on *M. tuberculosis* was comparable to isoniazid. However, it was possible that this partition and isoniazid could likely exert higher cidal activity on the lag phase cells over a longer exposure period and that its bactericidal activity was time-dependent. This possibility should be considered for future investigations. The high and rapid killing rates of the n-hexane partitions of *C. speciosus* and *T. coronaria* on the tubercle cells indicate that these plant partitions could contain effective anti-TB agents with high bactericidal capacity. Anti-TB agents with sterilising activity by killing all remaining viable bacilli are desirable to prevent relapse and hence, reduce the risk of resistance development [9]. It should be noted that these two partitions also exhibited good MIC (100 μg/mL), and MBC (100–200 μg/mL) values, which were evaluated on the mycobacterial cells using inoculum at log phase of growth. Hence, the results in this study also indicate that these plant partitions were effective against the mycobacterial cells during both lag and log phases of growth.

Observation under SEM showed that majority of untreated control tubercle cells on Day 3 of growth had smooth surface and well-defined rigid shape of different morphologies indicating that they were healthy and viable. The presence of some cells with wrinkled surface could be the leftover old cells in the inoculum. The cell population had been shown to undergo log phase of growth on Day 3 (Fig. 3). The exponential growth stage was supported by the presence of many bacilli with constrictions or septa in the control cells, which indicate that these cells were dividing and undergoing active proliferation. According to previous reports [48, 49], the septa could be formed through an inward growth of cytoplasmic membrane and cell wall materials, which eventually closed off the cytoplasmic compartments of each daughter cell. Finally, part of the peptidoglycan that held the two cells together was hydrolysed to physically separate the cells [50, 51]. The curve or V-shape of the mycobacterial bacilli could be caused by post-fission snapping movements as suggested by related studies [52–54]. In the present study, these V-forms were probably temporal structures since very few of these cells were observed, which agreed with the observations made by a previous study [52] on the electron microscopy analysis of *M. tuberculosis* cell division. The study also reported similar observation of the presence of external ridges at the proximal ends of the mycobacterial cells and suggested that these ridges could be the original site of division. After the two daughter cells split, the site of division could continue to grow, forming a nub at the end of the cell. Concerning the parallel-conjoined cells in pairs, an early report [55], suggested that they could be formed due to the growth of a daughter cell budding from the side of the mother cell and its subsequent growth along the side of the mother cell before splitting into two separate cells. In the present study, the damaging effects of isoniazid and the plant partitions on the cellular integrity of *M. tuberculosis* were shown by the observation of a variety of morphologies with evidences of structural changes. Majority of the treated cell population were degraded cells with wrinkled, ragged, and shrunk surfaces indicating dilapidation of the overall cell structure. The presence of tiny holes and dents on the cell surfaces could be due to the lethal effects of the plant partitions and isoniazid, which caused the cellular contents or cytoplasmic materials to leak out from the cells. Some cells that appeared like empty shells with hollow ends could be the eventual effect when all the cytoplasmic materials extruded out, indicating loss of viability. In a related study [29], it was observed that the extrusion of cellular contents of *M. tuberculosis* H37Ra cells exposed to isoniazid eventually caused the cell structure to collapse. The report suggested that the extrusion of cellular contents or cytoplasmic materials from the cells could be the rationale for the formation of the “web-like” materials, which were also observed around the cells in this study. These in turn might cause the cells to clump together, as shown by the presence of patches of amorphous mass of cell debris. These extracellular materials could probably be alkali-extractable polysaccharides being released into the external medium by treated mycobacterial cells as reported by an early study [56] on the effects of isoniazid. Alternatively, according to another study [57], the materials could be composed mainly of proteins, which were over-secreted following the action of the same drug. The study suggested that this effect caused the cells to shrink and eventually, these deformed cells aggregated into an amorphous mass of cell debris. The last cellular feature observed in this present study was slight swelling of the poles of some treated cells, which made them appear as club-shape and bulges. This feature was also observed in a related study [58]. The study suggested that it could be attributed to the ability of the mycobacterial bacilli to survive environmental hardship or the morphological changes probably started at the bacterial poles, which represent the weaker regions of the growing cells.

## Conclusions

The n-hexane partition of the plant materials exhibited the highest inhibitory activity against *M. tuberculosis* H37Rv indicating that the active phytochemical constituents could be of lipophilic nature. This indication was supported by the identification of many lipophilic constituents in the partitions using GC-MS analysis. The high killing rate over time shown by the n-hexane partitions of *C. speciosus* stem-flower and *T. coronaria* leaf indicates that the active constituents in these plant partitions could serve as sterilising agents against the mycobacterial cells during both lag and log phases of growth. The mycobacteriostatic activity of n-hexane partition of *C. citratus* stem-rhizome was comparable to isoniazid and its mycobactericidal activity could be time-dependent. Finally, the lethal effects of all the n-hexane plant partitions altered the normal mycobacterial cellular structure and caused cell lysis, thus, prevented proliferation of the cells.

## Abbreviations

ADC: Albumin, dextrose and catalase
ATCC: American Type Culture Collection
CFU: Colony forming unit
CO_2_: Carbon dioxide
DMSO: Dimethyl sulphoxide
HMDS: Hexamethyldisilazane
MB: Middlebrook
MBC: Minimum bactericidal concentration
MIC: Minimum inhibitory concentration
OADC: Oleic acid, albumin, dextrose and catalase
PBS: Phosphate-buffered saline
SEM: Scanning electron microscope
TB: Tuberculosis
WHO: World Health Organisation
